# Effect of self-assembling peptide P_11_-4 on orthodontic treatment-induced carious lesions

**DOI:** 10.1038/s41598-020-63633-0

**Published:** 2020-04-22

**Authors:** A. Welk, A. Ratzmann, M. Reich, K. F. Krey, Ch. Schwahn

**Affiliations:** 1 Dental school of the University Medicine Greifswald, Department of Restorative Dentistry, Periodontology, Endodontology, Preventive and Pediatric Dentistry, Greifswald, Germany; 2 Dental school of the University Medicine Greifswald, Department of Orthodontics, Greifswald, Germany; 3 Dental school of the University Medicine Greifswald, Department of Prosthodontics, Greifswald, Germany

**Keywords:** Fixed appliances, Fixed appliances, Minimal intervention dentistry, Minimal intervention dentistry, Randomized controlled trials

## Abstract

This study aimed to evaluate the effect of self-assembling peptide P_11_-4 (SAP) in the therapy of initial smooth surface caries (white spot lesions, WSL) following orthodontic multibracket treatment. Twenty-three patients (13f/10m; average age 15.4 years) with at least two teeth with WSL were recruited for the randomised controlled clinical trial with split-mouth design. In opposite to the control teeth, the test teeth were treated with SAP on Day 0. The primary endpoint was the impedance measurement of WSL using customised tray to ensure reproducibility of the measurement location. The secondary endpoint was the morphometric measurement of WSL using a semi-automated approach to determine the WSL size in mm^2^. Treatment effects were adjusted for site-specific baseline values using mixed models adapted from the cross-over design. Test WSL showed a mean baseline impedance value of 46.7, which decreased to 21.1, 18.4, and 19.7 after 45, 90, and 180 days, respectively. Control WSL showed a mean baseline value of 42.0, which decreased to 35.0, 29.5, and 33.7, respectively. The overall treatment contrast was −13.7 (95% CI: −19.6 – −7.7; *p* < 0.001). For the secondary endpoint, the test WSL size decreased from 8.8 at baseline to 6.5 after 180 days. The control WSL decreased from 6.8 to 5.7, respectively. The related treatment contrast was −1.0 in favour of test WSL (95% CI: −1.6 – −0.5; *p* = 0.004). The treatment of initial carious lesions with self-assembling peptide P_11_−4 leads to superior remineralisation of the subsurface lesions compared with the control teeth.

## Introduction

Orthodontic treatments with fixed multibracket appliances hindering oral hygiene, support plaque accumulation, and caries progression^1,2^. These orthodontic treatment-induced carious lesions are typically visible first as so-called white spot lesions (WSL) on the buccal surface of the tooth outlining the brackets^3–6^.

Modern treatment concepts for caries emphasise tooth preservation and remineralisation concepts especially for initial non-cavitated carious lesions, in order to hinder or to delay the first invasive intervention, meaning destruction of the natural tooth structure^7^.

Unique for buccal WSL is the addition of an aesthetic component to the cariological issue^3,8^. Fluorides prevent the formation of so-called white spot lesions (WSL) but have shown little effect on the reduction of existing WSL^9–11^. As their effect is restricted to the outer surface layer of the enamel (i.e. top 50 µm) and does not promote the remineralisation throughout the demineralised lesion body. The WSL persist visually almost unchanged^12–14^. Other remineralisation agents, often based on calcium phosphate, have been investigated, but could not show clinically significant advantage over fluoride^10,11,15–17^.

As a consequence, new treatment approaches have been called for and biomimetic mineralisation seems to be one promising possibility^18–22^. However, the only clinically available products at present are based on the self-assembling peptide P_11_-4 (SAP P_11_-4)^21^.

The mode of action for the treatment of initial caries with SAP P_11_-4 is as follows^23,24^. Monomeric P_11_-4 diffuses into the subsurface lesion, self-assembles into fibres to form a 3D-matrix and attracts calcium-ions from saliva and templates the formation of hydroxyapatite crystals, thus supporting the natural remineralisation mechanism driven by saliva.

Clinically, SAP P_11_-4 has previously been investigated on buccal lesions. A first-in-man trial, mostly on inactive lesions, demonstrated regression of the WSL size and a trend toward remineralisation^25^. Randomised clinical trials could show superior regression of the lesion as judged by morphometric analysis compared with both placebo^26^ and/or fluoride varnish^26,27^. As neither of the previous clinical trials investigated the effect of SAP P_11_-4 following orthodontic treatment, it can be assumed that the lesions investigated in those clinical trials were mostly inactive^25–27^.

Recently, the caries activity status of a lesion has become a new focus in cariology^28^.

Orthodontic WSL are of particular interest for clinical investigations of caries in this respect, as they are assumed to be active until debonding of the brackets, whereupon they become inactive^4,8^ and good accessible for several measurement methods^16,29^.

Therefore, the present split-mouth study investigated the effect of SAP P_11_-4 in addition to the conventional treatment of early carious lesions after debonding of orthodontic brackets. The primary endpoint was the impedance measurement^29–31^. Moreover, a semi-automated approach to measure the WSL size was used as done in other clinical studies on SAP P_11_-4^25–27^.

## Results

### Baseline characteristics

The mean duration of the orthodontic treatment with fixed appliances was 27 months (min/max 13/39). Of the 23 recruited patients (13f 10 m; mean age 15.4 years), 21 could be analysed in mixed models (12f/9 m; mean age 15.3 years). One patient showed established dentin caries (primary endpoint = 100) on both teeth after 30 days and cavitated lesions after 90 days. The related four values of the primary endpoint after 90 and 180 days were set to 100. No further cavitated lesions were observed. The QHI at t0 was 2.2/2.2 (test/control teeth).

### Impedance measurement of white spot lesion (primary endpoint)

Both test and control teeth exhibited a substantial decrease of impedance readings throughout the study period (Table 1). Control teeth showed a mean baseline value of 42.0 at day 0, which decreased to 35.0 at day 45, 29.5 at day 90, and 33.7 at day 180. Test teeth showed a mean baseline value of 46.7 and exhibited a markedly larger decrease to 21.1 (day 45), 18.4 (day 90), and 19.7 (day 180).

**Table 1:** Primary endpoint: Impedance measurement in test and control teeth (N = 21, split-mouth design, 122 observations in 2 sites or teeth at 3 time points).

	Observed values		Estimated values (adjusted for baseline values)			
	Test Group	Control Group	Test Group	Control Group	Treatment effect	
			Mean ± SE	Mean ± SE	Difference (95% CI)	*p* value
Baseline						
Mean ± SD	46.7 ± 16.9	42.0 ± 15.7				
Median (IQR)	50 (44–52)	47 (35–50)				
Day 45						
Mean ± SD	21.1 ± 23.2	35.0 ± 23.4	20.7 ± 5.1	35.4 ± 5.1	−14.7 (−22.8 – −6.6)	0.001
Median (IQR)	14 (7–23)	32 (17–51)				
Day 90						
Mean ± SD	18.4 ± 22.3	29.5 ± 25.0	18.2 ± 5.0	29.8 ± 5.0	−11.7 (−19.4 – −3.9)	0.005
Median (IQR)	10 (7–21)	19 (9–47)				
Day 180						
Mean ± SD	19.7 ± 24.2	33.7 ± 25.5	19.4 ± 5.4	34.0 ± 5.4	−14.6 (−24.5 – −4.8)	0.007
Median (IQR)	10 (5–27)	35 (10–49)				
Baseline vs						
Day 45						
Mean ± SD	25.5 ± 23.3	7.0 ± 21.7				
Median (IQR)	30 (18–43)	15 (−1–21)				
Day 90						
Mean ± SD	28.1 ± 25.1	12.2 ± 22.9				
Median (IQR)	36 (16–43)	14 (−4–32)				
Day 180						
Mean ± SD	26.8 ± 28.0	8.0 ± 27.4				
Median (IQR)	36 (17–45)	10 (1–28)				

According to the manufacturer impedance values correspond to the following:

Sound: 0 = sound; 1–20 = Sound enamel, caries at the very outer enamel; Enamel caries 21–30 = caries in the outer 1/3 of the enamel; 31–50 = caries in the middle 1/3 of the enamel; 51–90 = caries in the inner 1/3 of the enamel; 91–99 = caries at the dentine enamel junction; 100 = established dentine caries.

Abbreviation: CI, confidence interval; IQR, interquartile range; SD, standard deviation; SE, standard error of the mean.

**Figure 1:**
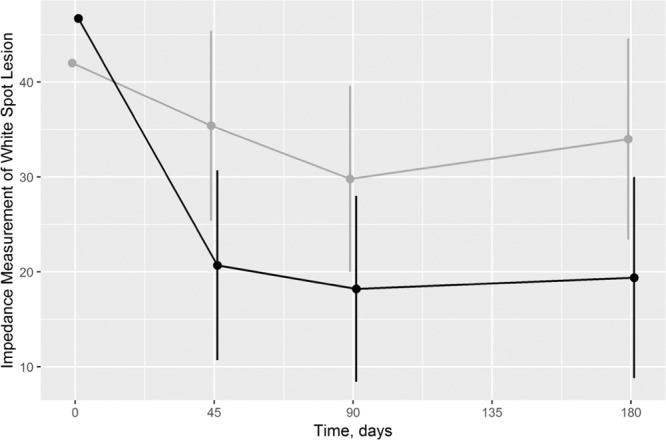
Impedance measurement of White Spot Lesion at different time points (black: test tooth/grey: control tooth). As “temporally and logically, a baseline cannot be a *response* to treatment, so baseline and response cannot be modeled in an integrated framework”^43^, baseline and response were graphed differently. Consequently, the response and the 95% CI are adjusted for baseline values^43^. *p* = 0.001, *p* = 0.005, and *p* = 0.007 for treatment differences after 45, 90, and 180 days, respectively.

The treatment effect was statistically significant (overall treatment contrast, which is the mean of the three single contrasts in Table 1: -13.7, 95% CI: −19.6 – −7.7; *p* < 0.001). More importantly, the global *F* test of no treatment effect was rejected (*p* = 0.003 for the test including one term for treatment and two terms for the interaction between treatment and time). After treatment, the difference between test and control tooth changed only slightly over time (*p* = 0.623; Fig. 1), yielding a large treatment effect of 43% after 180 days (14.6/34.0 in Table 1), which is usually considered clinically relevant. Robust analyses using the means of the three time points after treatment confirmed results of the mixed model in observed and imputed data with and without excluding dropouts (20, 21, and 23 subjects, respectively; treatment differences −13.1 (95% CI: −18.8 – −7.4), −13.3 (95% CI: −20.2 – −6.3), and −12.8 (95% CI: −22.4 – −3.2), respectively; for the “missing not at random” scenario by adding 50 to the treatment site of the three dropouts: −9.7 (95% CI: −18.9 – −0.5)).

### Morphometric measurement of white spot lesion (secondary endpoint)

**Figure 2:**
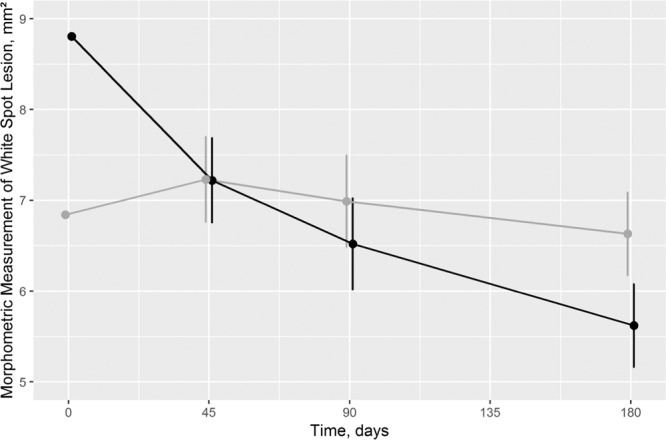
Morphometric measurement of White Spot Lesion Size in mm^2^ at different time points (black: test tooth/grey: control tooth). As “temporally and logically, a baseline cannot be a *response* to treatment, so baseline and response cannot be modeled in an integrated framework”^43^, baseline and response were graphed differently. Consequently, the response and the 95% CI are adjusted for baseline values^43^. *p* = 0.969, *p* = 0.137, and *p* = 0.004 for treatment differences after 45, 90, and 180 days, respectively.

After treatment, the decrease in size according to semi-automated morphometric measurements was more pronounced in test teeth (*p* = 0.030 for the interaction between treatment and time; Fig. 2). After 180 days, the difference between test and control group was statistically significant in mixed model analysis although the 95% CI was also consistent with a weak to moderate effect (Table 2). The global test of no treatment effect was rejected (*p* = 0.013).

**Table 2:** Secondary endpoint: Morphometric measurement (mm^2^) in test and control teeth (N = 17, split-mouth design, 94 observations in 2 sites or teeth at 3 time points).

	Observed values		Estimated values (adjusted for baseline values)			
	Test Group	Control Group	Test Group	Control Group	Treatment effect	
			Mean ± SE	Mean ± SE	Difference (95% CI)	*p* value
Baseline						
Mean ± SD	8.8 ± 7.8	6.8 ± 5.1				
Median (IQR)	8.6 (2.0–11)	6.7 (2.8–9.6)				
Day 45						
Mean ± SD	8.1 ± 7.3	6.2 ± 4.7	7.2 ± 0.24	7.2 ± 0.24	0.0 (−0.6 – 0.6)	0.969
Median (IQR)	6.0 (1.9–11)	5.9 (2.4–9.9)				
Day 90						
Mean ± SD	7.5 ± 7.0	6.1 ± 4.6	6.5 ± 0.26	7.0 ± 0.26	−0.5 (−1.1 – 0.2)	0.137
Median (IQR)	5.4 (2.4–10)	5.4 (2.5-9.1)				
Day 180						
Mean ± SD	6.5 ± 6.5	5.7 ± 4.4	5.6 ± 0.24	6.6 ± 0.24	−1.0 (−1.6 – −0.4)	0.004
Median (IQR)	5.0 (0.5–11)	4.9 (2.4–9.4)				
Baseline vs						
Day 45						
Mean ± SD	1.2 ± 1.0	0.9 ± 1.1				
Median (IQR)	0.9 (0.4–1.9)	0.4 (0.1–1.3)				
Day 90						
Mean ± SD	1.8 ± 1.4	1.1 ± 0.9				
Median (IQR)	1.7 (0.6–2.8)	0.9 (0.3–1.7)				
Day 180						
Mean ± SD	2.7 ± 1.7	1.5 ± 1.3				
Median (IQR)	2.5 (1.3–3.8)	1.2 (0.4–2.5)				

Missing values explain discrepancies between differences of observed values and calculated changes from baseline (see results).

Abbreviation: CI, confidence interval; IQR, interquartile range; SD, standard deviation; SE, standard error of the mean.

Missing values were also dealt with by multiple imputation, including sensitivity analysis for missing data when true values are systematically lower or higher (nonignorable non-response). Both WSL of one patient were too small to be measured semi-automatically. The two cavitated lesions were not measurable after 90 and 180 days. These paired nonignorable non-responses are levelled off by the split-mouth design. Missing values, for which ignorable non-responses were assumed, occurred for a total of four subjects. One of the two patients who were excluded in mixed models for missing baseline values could be re-included in robust analysis, resulting in 15 patients available for complete case analysis. Robust analysis of contrasts between the first and third time points (-t_1_ + t_3_ to model the interaction) confirmed the result of the mixed model by using complete and imputed data without dropouts (15 and 21 subjects, respectively). The treatment effect became uncertain if “ignorable non-response” did not hold in sensitivity analyses after multiple imputation and by including dropouts (23 subjects).

## Discussion

Our trial raised comparable parameters as the previously reported trials^25–27,32^ but differs from the other trials on buccal caries, as active carious lesions (clinically visible as rough, chalky and matte surface) were treated, and not predominantly inactive ones (clinically visible as a white spot under a hard, shiny surface)^25–27^. Both the impedance values and morphometric measurements showed favourable WSL reduction results for the test group.

The greater difference between test and control lesions, however, occurred in the impedance values compared with that in the morphometric measurements, thereby confirming that SAP P_11_-4 acts predominantly by regenerating the mineral structure of the enamel of the lesion body and not just the surface^23,24^. Moreover, at the last study visit at day 180, the impedance values for test lesions indicated regression of caries into very outer enamel, whereas the impedance values for the control lesions indicated still caries throughout the whole enamel layer^31,33^, which agrees with previous data that fluoride (in the present study as fluoride-based professional prophylaxis paste and home care toothpaste) only acts in the top 30-50 µm of the enamel surface^13,14,34^.

In recent years, caries activity has become a central topic within cariology, as activity defines the clinical need and opportunity to intervene in the decay process. Active carious lesions, as treated in the present study, have open pores allowing communication between the subsurface carious lesion body and the oral cavity. From the lesion body, calcium and phosphate ions can diffuse out (demineralisation) or in (remineralisation). In a similar fashion, the open pores allow SAP P_11_-4 to diffuse into the lesion body to support remineralisation and thus regression of the tooth decay.

The active carious lesions in the conventional treatment control exhibited spontaneous regression of the measured WSL size over the study period until day 180^3,8^, which is comparable to literature data^27,29^. However, SAP P_11_-4 enhanced the remineralisation to a higher decrease of the measured WSL size.

The present clinical trial is not without shortcomings. The possibility of random error because of the relatively small sample size. Second, the trial was run within a university setting and the patients were Caucasians, thereby limiting the generalizability of the findings. Third, information bias could have influenced our results. The morphometric assessment represents only a part of the clinical situation because it is related only to the visible tooth surface. Critical clinical assessment factors such as the hardness and gloss of the lesion could not be taken into account as they could not be measured reliably within an *in vivo* study. It would be interesting to measure the enamel hardness induced by SAP P_11_-4, and compare those to the previously reported *in vitro* microhardness results^34,35^.

However, there are also several strengths. First, the source population and the chosen inclusion criteria ensured that active carious lesions, not inactive ones, were investigated. Second, the split-mouth design is appealing since “each participant acts as their own control”^36^. By this favourable control mechanism, the split-mouth design is related to counterfactual queries (would a subject have no caries had the subject been treated with SAP given that the subject has in fact caries and is not treated with SAP), whereas the usual randomised clinical trial in parallel-groups is merely related to intervention queries (would a subject have no caries if we make sure that the subject is treated with SAP)^37^. Therefore, the split-mouth design is clearly superior to a parallel group design (unless in identical twins) if the study design assumptions hold^38^. As SAP P_11_-4 acts solely on tooth level^23,24^, it is biologically well justified to assume the absence of any cross-contamination or carry-across effect from one site to another^39^. Note that the theory of causality^37^ has been rapidly developed since the split-mouth design was criticized for statistical reasons in 1990^40^. Third, the split-mouth design was very efficient because the within-patient correlation was clearly different from zero (*p* = 0.61 for the primary endpoint, *p* = 0.72 for the secondary endpoint). For the primary and secondary endpoint, the sample sizes of n = 21 and n = 17 in our split-mouth design correspond to total sample sizes of 108 and 118 patients in a parallel group design, respectively^39^. Fourth, three measurements of the primary endpoint were taken to increase reliability. Fifth, the duration of the trial worked for both endpoints. It was long enough to gain important insights into the mechanism of the treatment, especially for the secondary endpoint. Finally, the statistical analysis is state-of-the-art^39,41–43^, follows rigorously the intention-to-treat principle by accounting for the uncertainty due to dropouts, and supports confidence intervals^44^.

Overall, our results not only agree with other clinical trials on the effectiveness of SAP P_11_-4^20,25–27^ but also show that SAP P_11_-4 is effective in the treatment of active WSL^26,27^ supporting as already mentioned the proposed mechanism of action within the subsurface lesion body^23,24^. Thus, SAP P_11_-4 treatment gets one step closer to a regeneration ad integrum, that would be the ultimate goal in healing carious enamel.

One of the reasons that the treated enamel does not return to full translucency is seen in the fact that the SAP P_11_-4-induced fibres support the formation of de novo hydroxyapatite crystals in a fan-type non-prismatic arrangement around the fibres^24,45^, which have another refractive index than the prismatic hydroxyapatite crystals produced by the Tomes processes from ameloblasts in the final stage of enamel deposition.

Although buccal carious lesions chosen in order to quantitatively assess the effect of the treatment are rare outside the orthodontics, the clinical significance of the results relates to the treatment of caries in general. Thus, the aesthetic shortcomings of the SAP P_11_-4 treatment^46^ are neglectable for almost all of the initial carious lesions as most clinically relevant caries develops in proximal or occlusal sites and if buccal lesions occur, they are mostly positioned on premolars and molars.

Professionals are trained to identify initial caries in any location, either visually, with caries diagnostics or on x-rays. Based on those assessments the clinician will decide on whether invasive restorative treatments are needed. Regenerative procedures should be considered whenever possible, in order to conserve natural tooth structure and function and to avoid or at least to delay as far as possible the entry into the conventional filling approach leading to ever larger fillings^47^. Bröseler *et al*. have shown that the SAP P_11_-4 treatment can be transferred into a practice setting^27^.

## Conclusion

The treatment of initial carious lesions with self-assembling peptide P_11_-4 leads to superior remineralisation of the subsurface lesions compared with the control teeth.

## Material and Methods

### Study design

**Figure 3:**
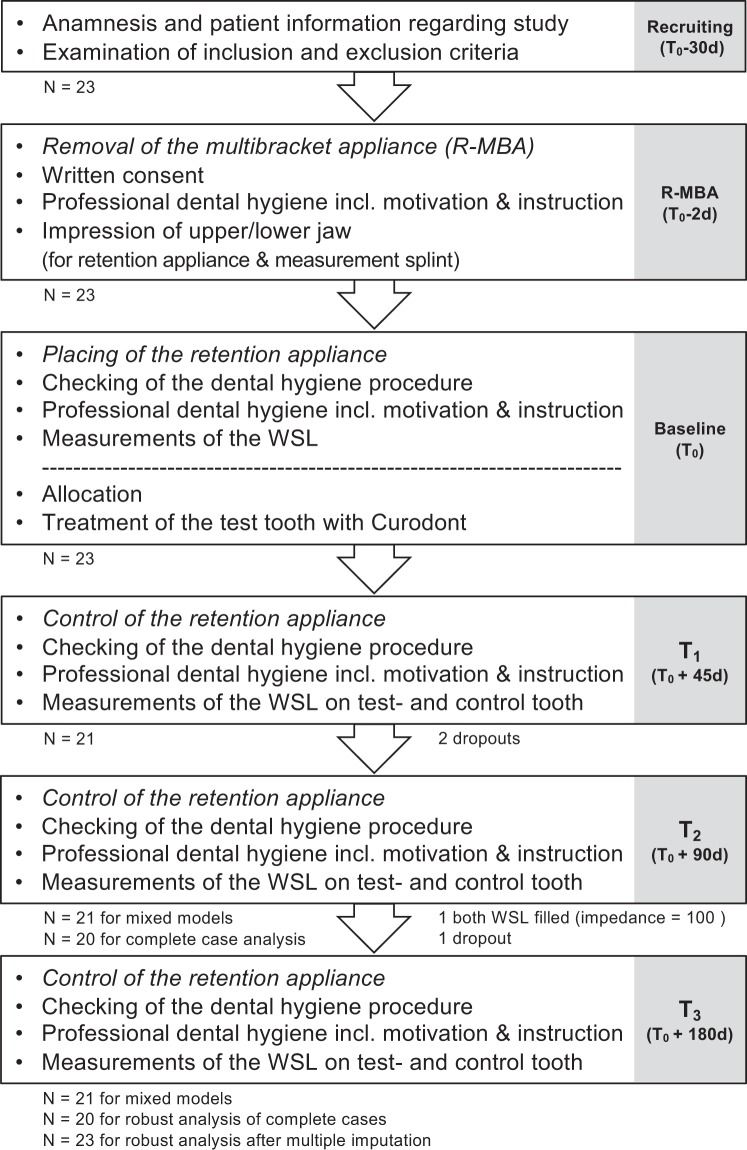
Patient Flow Chart.

The clinical trial was designed and performed as a split-mouth conventional treatment-controlled trial investigating the effect of the treatment of buccal carious lesions following orthodontic treatment by using SAP P_11_-4 as add-on with the conventional treatment control. Clinical study procedures were performed parallel to regular appointments at the Orthodontic Department of the University Medicine Greifswald (Fig. 3) according to the Declaration of Helsinki and in compliance with ISO 14155:2012. Approval for all clinical procedures and the trial was obtained by the ethical committee of the University of Greifswald (Code BB 99/12, date of approval: 28^th^ August 2012).

The clinical trial was registered in the German Database for clinical trials (DRKS00016501, date of registry: 30^th^ January 2019).

### Patient and study tooth selection

Twenty-three patients (13f/10 m; average age 15.4 years) with at least two WSL after removal of fixed orthodontic appliances could be recruited into the trials from the Orthodontic Department of the University Medicine Greifswald and treated by the Restorative Department. The written informed consent was obtained from every participance or from a parent of every participance under 18 years prior to any study-related procedures two days before baseline (Fig. 3).

Patients had to fulfil all the following selection criteria:

Inclusion

- At least two active carious lesions around the bracket area with a rough, chalky and matte surface;
- Size and form of the active carious lesion: The carious lesion must be fully visible and assessable and accessible;
- Able and willing to observe good oral hygiene throughout the study;
- ≥20 teeth and a BP score of <3;
- Age ≥12 years and ≤18 years;
- Willing and able to attend the on-study visits;
- Willing and able to understand all study-related procedures;
- Written informed consent before participation in the study.
  Exclusion
- Pre-treated WSL;
- Tooth with another carious lesion apart from the ones developed during orthodontic treatment;
- Evidence of tooth erosion; History of head and neck illnesses;
- Any pathology or concomitant medication affecting salivary flow;
- Concurrent participation in another clinical trial.

### Randomisation and blinding

Within each subject, the treatment was randomly allocated to a site by flipping a coin. For three subjects having had test and control teeth in the same quadrant, the more anterior tooth was assigned to the opposite site. Based on our add-on study design, the investigator and patient were not blinded during applying SAP on the tooth, however, outcome assessor and statistician (except for sensitivity analysis after multiple imputation described below) were blinded.

### Study treatment procedures (besides the regular orthodontic treatment)

At the Day 0 visit, all teeth were cleaned with a non-fluoridated tooth cleaning paste (Clean Polish, Kerr, Germany), (Fig. 3).

Further, the patients received an electrical tooth brush (Oral-B Pro 1000 Precision Clean, P&G, Germany) and an instruction in it. After that, general patient assessment was recorded prior to allocation of the treatment.

“Each participant acts as their own control”^36^. In opposite to the control teeth, which got no further treatment on Day 0, the test teeth were prepared according to the instruction of the use for the SAP P_11_-4 product (Curodont Repair, Credentis, Windisch, Switzerland). The test teeth were cleaned with 2–3% NaOCl, rinse with H_2_O; etched with 35% Phosphoric acid for 20 sec (Ultra-Etch, Ultradent Products Inc., USA), rinsed with H_2_O and air-dried. After cleaning, the SAP P_11_-4 solution was applied to the WSL and left in place for 5 minutes.

The patients were strongly encouraged to use fluoride toothpaste (at least 1450 ppm Fluoride) twice daily.

At every follow-up visits (Day 45, Day 90 and Day 180) parallel to regular orthodontic control appointments, all teeth (including test tooth and control tooth) were cleaned with a fluoridated prophylaxis paste (Flairesse 12.300 ppm Fluoride, DMG, Germany) as at 2 days before baseline (Day 0). Oral hygiene instructions were provided at every study visit (Fig. 3).

### Outcomes

**Figure 4:**
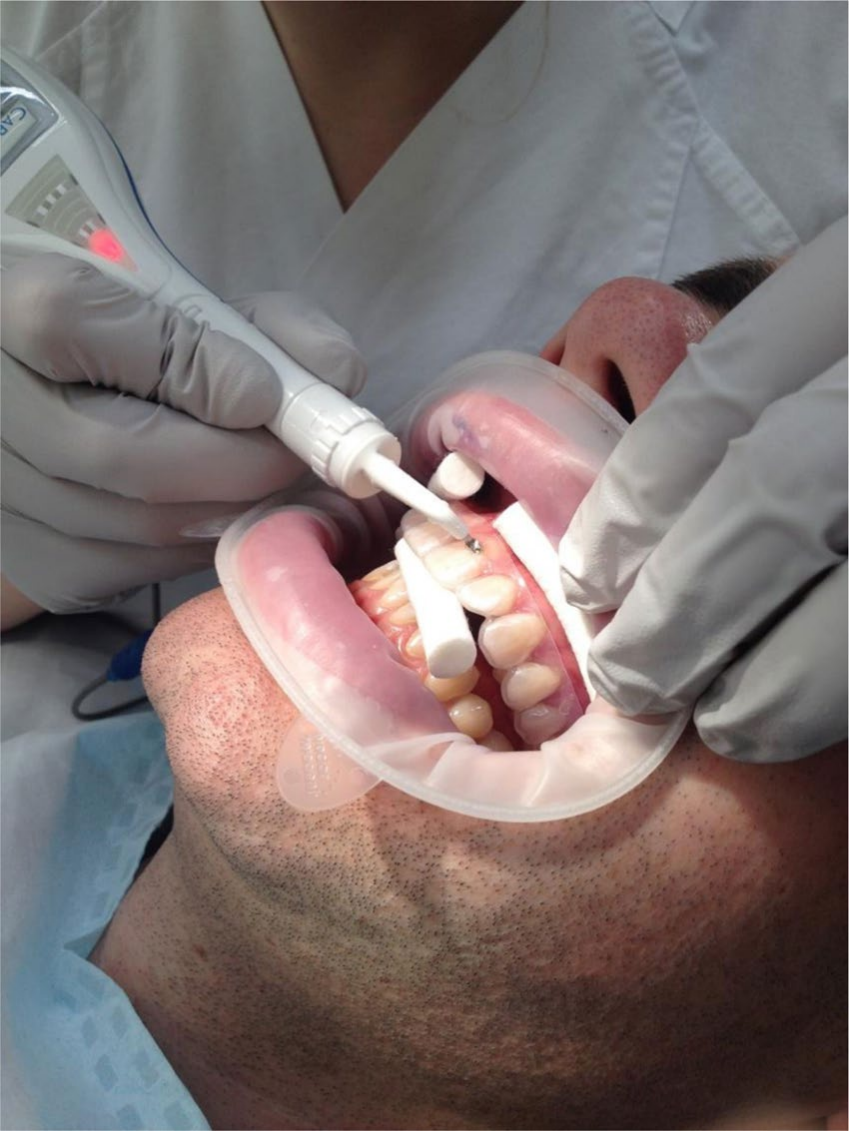
Image of Impedance measurement of WSL with CarieScan Pro (Orangedental/Biberach/Germany).

Primary endpoint of the study was initial carious lesions as measured by the impedance (CarieScan, Orange Dental, Biberach, Germany)^29–31^. Before measurements, the device was checked and handled according to the manufacturer’s instructions. The area was kept dry by optragate (Ivoclar Vivadent, Ellwangen, Germany) and the tooth surface air-dried for 6 seconds. The impedance measurements were taken three times and the internal CarieScan values were averaged for each time point. To ensure reproducibility of the location of the WSL throughout the study, customised trays were prepared, and holes were drilled at the place of the WSL fitted to the nozzle of the diagnostic. The measurements were performed with the tray in place to ensure that readings were taken at the same place (Fig. 4).

**Figure 5:**
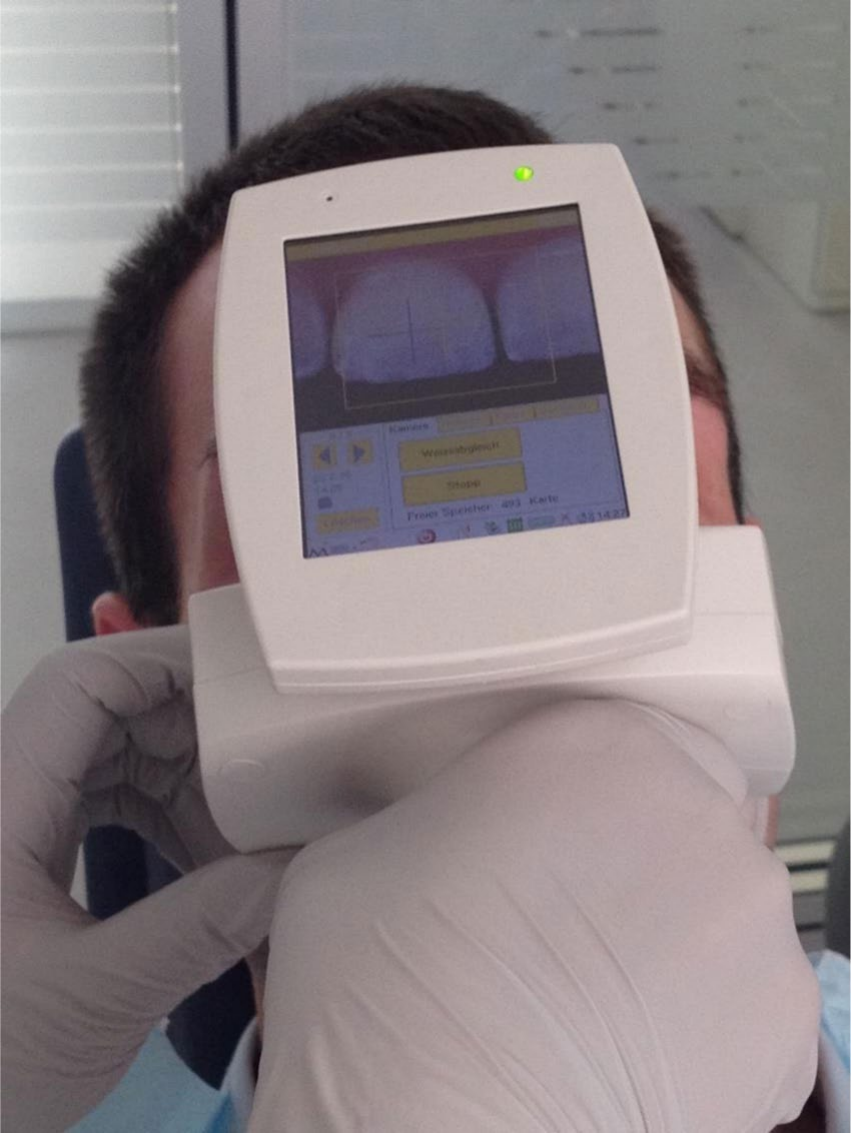
Overview image of Morphometric measurement of WSL with Shadepilot (DeguDent/Hanau/Germany).

**Figure 6:**
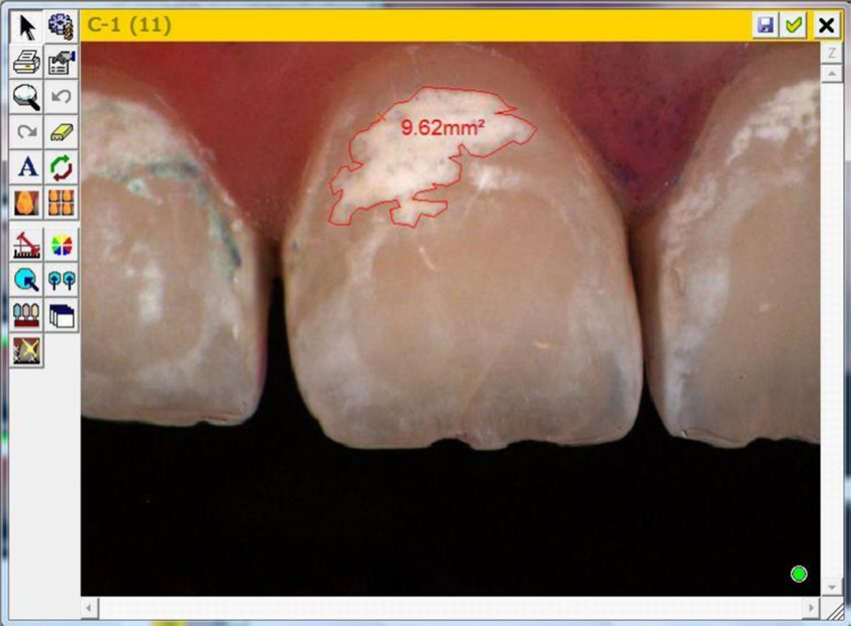
Computer Screen image of Morphometric measurement of WSL with Shadepilot (DeguDent/Hanau/Germany).

The secondary endpoint (WSL size) throughout the study were evaluated morphometrically via Shadepilot (DeguDent, Hanau, Germany) (Fig. 5). In order to ensure reproducibility of the measurement, a representative and isolated area of the WSL on the study tooth was selected and its size semi-automatically determined as follows: A buccal tooth area was selected on the measuring device, the WSL area within the area calculated by the device, and refined, if necessary, by the blinded assessor. For clarity: Not necessarily the whole affected area of the study tooth was taken into account but only clearly definable WSL (Fig. 6).

### Sample size estimation

At the time of designing the study previous clinical data were not available whereas the SAP treatment was new. The experience over the last years supports that “In practice, for many trials, it is unlikely that there will be data to support a realistic estimate” of the within-subject correlation^36^. This is especially true in dentistry, where data from at least three levels (subject, jaw, and tooth) are required to account for within-subject variability. Thus, “it is a more sensible approach to sample-size determination to have a look at what sort of trial has been run in the past in a particular area and see what sort of inferences were possible rather than going through some complicated power calculation: often this is no more than a ritual”^48^. This is what was done; for the primary endpoint, which is continuous, at least 22 participants were aimed for^49,50^. Note that inferences from confidence intervals as presented herein are more appropriate than inferences from p-values^44,51^.

### Statistical analysis

Replacing “period” by “site”, the split-mouth design was analysed as cross-over design^39^. The repeated measurements were examined in mixed models for random subject effects and in robust analyses for the two-treatment design^52^. Treatment, time, the interaction between treatment and time, site-specific baseline values using restricted cubic splines with 3 knots, site, and the interaction between site and time were fixed factors. In addition, subject and site were included as hierarchical random factors, and time was modeled as repeated factor by three variance and three unstructured covariance terms within site, yielding a total of seven random factors^52^. The Kenward-Roger method^41^ using the observed information matrix was applied to correct for small-sample inference. Mixed models can deal with imbalanced or missing data if the “missing at random” assumption holds^43^. The complex mixed models were analysed using Stata software (release 14.2, Stata Corporation, College Station, TX, USA) and checked using SAS software (release 9.4, Cary, NC, USA). In robust analyses of the direct treatment effect (not including baseline values), the two-sample *t*-test was used for differences in linear combinations of repeated measurements between groups defined by the site of treatment^52^. To cover the uncertainty due to dropouts, multiple imputations were generated by fully conditional specification using R software^43^. Scenarios other than “missing at random” were examined in sensitivity analysis after multiple imputation.

## Data Availability

The datasets of the current study are available from the corresponding author on request.
